# Chinese whispers: COVID-19, global supply chains in essential goods, and public policy

**DOI:** 10.1057/s42214-020-00075-5

**Published:** 2020-11-04

**Authors:** Simon J. Evenett

**Affiliations:** 1 Department of Economics, University of St. Gallen, St. Gallen, Switzerland; 2 SIAW-HSG, Bodanstrasse 8, 9000 St. Gallen, Switzerland

**Keywords:** China, COVID-19, global value chains, protectionism, trade

## Abstract

If taken at their word, senior policymakers in the major economic powers have drawn adverse conclusions concerning the performance of cross-border supply chains during the first 6 months of the COVID-19 pandemic. That such supply chains often implicate China, the origin of the pandemic, has also led to claims that trading partners have become too dependent on Chinese supplies. This in turn has led to policy interventions designed to reconfigure supply chains, which if adopted broadly could revise the terms upon which international business operates. A critical evaluation of this policymaker assessment is presented, based on near-time monitoring of medical and food trade disruption induced by government policy, on fine-grained trade data on the pre-pandemic international sourcing patterns of medical goods and medicines by France, Germany, the United Kingdom, and the United States, on statements from U.S. government health experts before and during the pandemic on the frequency and sources of medicine shortages, and on the U.S. Food and Drug Administration’s latest evidence on the causes of medicine shortages in 2020. Such evidence vitiates the adverse conclusions mentioned above, but raises important questions about the factors that determine policy towards international business during a time of intensifying geopolitical rivalry.

## INTRODUCTION

On January 30, 2020, the Director-General of the World Health Organization declared that the novel Coronavirus outbreak was a “public health emergency of international concern,”, which was the organization’s highest level of alert and longhand for a global pandemic. What began as a public health crisis soon had significant economic and commercial consequences, some of which were induced by the very public health and macroeconomic policy responses taken to confront the pandemic.

The shortages of personal protective equipment (PPE) that came to light in the first 6 months of 2020 have led many policymakers to conclude – if their public statements are anything to go by – that existing cross-border supply chains are no longer fit for purpose in essential goods sectors. Assertions have been made that an unhealthy overdependence on China has arisen, in short that globalization has gone too far.^1^

Governments of major economies have contemplated repatriating supply chains through a variety of incentives or by restricting access to public sector contracts to local producers. Independently of government, corporate executives and their advisers have begun to reconsider the configuration of their international supply chains, couching these initiatives in terms of “building resilience,” “diversification,” etc. (McKinsey Global Institute, 2020; Mirodout, 2020).

What is the intellectual significance of these developments for scholars of international business, economics, and political economy? To appreciate what is at stake, perhaps it is best to start by referring to another field of intellectual endeavor. In his magisterial history of pandemics and their societal consequences, the Yale historian Frank Snowden advanced the hypothesis that:

epidemics are not an esoteric subfield for the interested specialist but instead are a major part of the ‘big picture’ of historical change and development. Infectious diseases, in other words, are as important to understanding societal development as economic crises, wars, revolutions, and demographic change (Snowden, 2019).

The fit, then, is evident for an academic journal committed to examining the contribution of international business to “grand challenges” (Buckley, Doh, & Benischke, 2017; Lundan, 2018; Van Assche, 2018). Putting the matter more narrowly, to what extent were cross-border supply chains part of the problem, part of the solution to the COVID-19 pandemic, or both? Given that the pandemic is not over, the goal in this paper is to shed light on factors known now that are relevant to answering this question.

In this paper, particular attention is given to the conclusions drawn by leading policymakers concerning the efficacy of cross-border supply chains in the first 6 months of the pandemic. If policies are adopted in line with these conclusions, then they could have profound implications for the incentives and constraints faced by international business as they organize their cross-border operations (Altman, 2020; Kobrin, 2020). This would not be the first time that policy shifted sharply to harness multinational business, as the research of Stephen Kobrin, Raymond Vernon, and others have shown (Aguilera, Henisz, Oxley, & Shaver, 2019; Boddewyn, 2016; Graham, 2000; Ghemawat, 2016, 2018; Kobrin, 2017, 2020; Vernon 1971, 1977, 1988).^2^

Although the focus of this paper is on the inter-relationship between public policy intervention and cross-border supply chain performance in the early phase of the pandemic, three important points of context are worth bearing in mind. First, current and potential future reconfiguration of cross-border supply chains is taking place in the context of enhanced rivalry between leading economic powers, of which the Sino–U.S. trade and technology war is one salient manifestation (Blustein, 2019; Davis & Wei, 2020; Evenett & Fritz, 2018; Petricevic & Teece, 2019). It is not for nothing that political scientists are rethinking their understanding of Economic Statecraft (Baldwin, 1985; Aggarwal & Reddie, 2020).

Second, cross-border commercial operations are likely being recast in light of the build-up since the Global Financial Crisis of policies seeking to influence cross-border flows of goods and services, investments, ideas, and workers (Evenett, 2019). By and large, policies introduced to favor local firms have been far more prevalent than policies leveling the commercial playing field for foreign rivals. It may have taken the U.S.–China trade war to lay to rest claims that the World Trade Organization’s rulebook acted as a serious constraint on government policy choice, but companies with substantial in-house capacity to monitor trends globally had already picked up of these shifts (as the example of General Electric shows, see Bhatia, Evenett, & Hufbauer, 2016). These trends were well underway before many governments turned towards populism and economic nationalism (Rodrik, 2018).

Third, even before the U.S.–Sino trade war, and certainly before the COVID-19 pandemic, there was growing evidence “hidden in plain sight” that businesses themselves were reconfiguring supply chains (McKinsey Global Institute, 2019). The factors responsible include rising wages in China, speed-to-market growing in importance as a competitive strength of firms, shifts in public policy that encourage sourcing locally or in regional trade partners, adoption of digital technologies and more generally “intangibles” playing a greater role, and a greater appreciation of the risks faced from operating supply chains over long distances or in less well known cultures and business climates. Advocacy of “near-shoring,” “localization,” and “produce where you sell” strategies preceded the COVID-19 pandemic.

The remainder of this paper is organized as follows. The next section outlines the trade policy changes in two so-called essential goods sectors, namely, food and medical supplies and medicines, and the attendant disruption to cross-border supply chains. The focus on these sectors is warranted given their salience in the media and their apparent influence on policymakers’ perceptions of the performance to date of cross-border supply chains during the pandemic. Indeed, according to the Global Trade Alert database, 38% of commercial policy interventions this year implicate these two sectors.

The third section begins by documenting the dissatisfaction with the operation of cross-border supply chains by senior policymakers in leading economies including the insinuation that the dependence on China for essential goods is too high and needs to be reduced. These claims are then critically evaluated using a range of evidence. The implications of this evaluation for the way in which governments determine the policies likely to confront international business in the years ahead is discussed in the concluding section.

As this outline implies, the approach taken in this paper places a premium on collecting and assessing relevant up-to-date evidence that can inform thinking. Bringing to the attention of the scholarly community relevant, perhaps overlooked evidence, some of which was specifically collected by the author and his collaborators, is one of the intended contributions of this paper.^3^ Those seeking fancy econometrics or parsing of theories may be disappointed. The Nobel Prize winner and founder of modern economic analysis, Paul A. Samuelson, made the following point: “…the first duty of an economist is to describe correctly what is out there: a valid description without a deeper explanation is worth a thousand times more than a clever explanation of nonexistent facts” (Samuelson, 1963). That statement is the lodestar of this paper.

## THE INITIAL TRADE POLICY RESPONSE TO THE PANDEMIC IN FOOD, MEDICAL SUPPLIES, AND MEDICINES, JANUARY–SEPTEMBER 2020

As COVID-19 spread, more governments began taking public health measures and restricting international travel including in some cases sealing borders. The former initiatives led to a surge in demand for many medical suppliers and medicines, raising fears about shortages. The disruption of cross-border shipments of goods also led to fears of food insecurity. Net importers of food and medical supplies worried that goods necessary to support basic living standards and health would not be delivered, even when contracted and paid for. For net exporters, concerns were raised that too many so-called essential goods were being exported and not enough held back for their own residents.

Given the large number of regional trade agreements (RTAs), and the global trade rulebook at the World Trade Organization (WTO), one might be tempted to conclude that the institutions and incentives were in place to deter disrupting trade in essential goods. In fact, the WTO rulebook has more extensive rules constraining the use of import restrictions than of export curbs, and the existing web of RTAs hardly fills in the gap. This rarely invoked lacunae in global economic governance was exposed once the pandemic began to spread beyond China, to the detriment of those firms engaged in international supply chains in the affected products.

An aggravating factor was the lack (initially at least) of any official monitoring of resort to export curbs. Without such information, policymakers and business decision-makers would have operated in factual vacuum, a situation ripe for fear dominating reason. Evidence on trade policy choices was assembled initially by the Global Trade Alert team based at the University of St. Gallen, and then in a joint initiative with the World Bank and European University Institute.^4^ That initiative sought to document all of the changes in export controls, import tariffs, import quotas, taxes on imported goods, and other relevant non-tariff barriers affecting global trade in food, medical goods, and medicines that were announced and implemented since January 1, 2020.^5^

By September 4, 2020, a total of 660 trade policy interventions in these essential goods sectors had been documented. A total of 459 such interventions implicate the medical goods and medicines sectors, while 238 implicate the food sector.^6^ Just under half of the interventions (328) restricted trade and 332 liberalized trade, implying that the media attention on export curbs told only part of the story. This initiative documented twice as many trade policy interventions as that of the International Trade Centre,^7^ one of the three international organizations that eventually began monitoring developments in this area.^8^ In what follows, developments in the medical goods and medicines sector are described first, then those in the food sector.

### Medical Goods and Medicines

**Figure 1:**
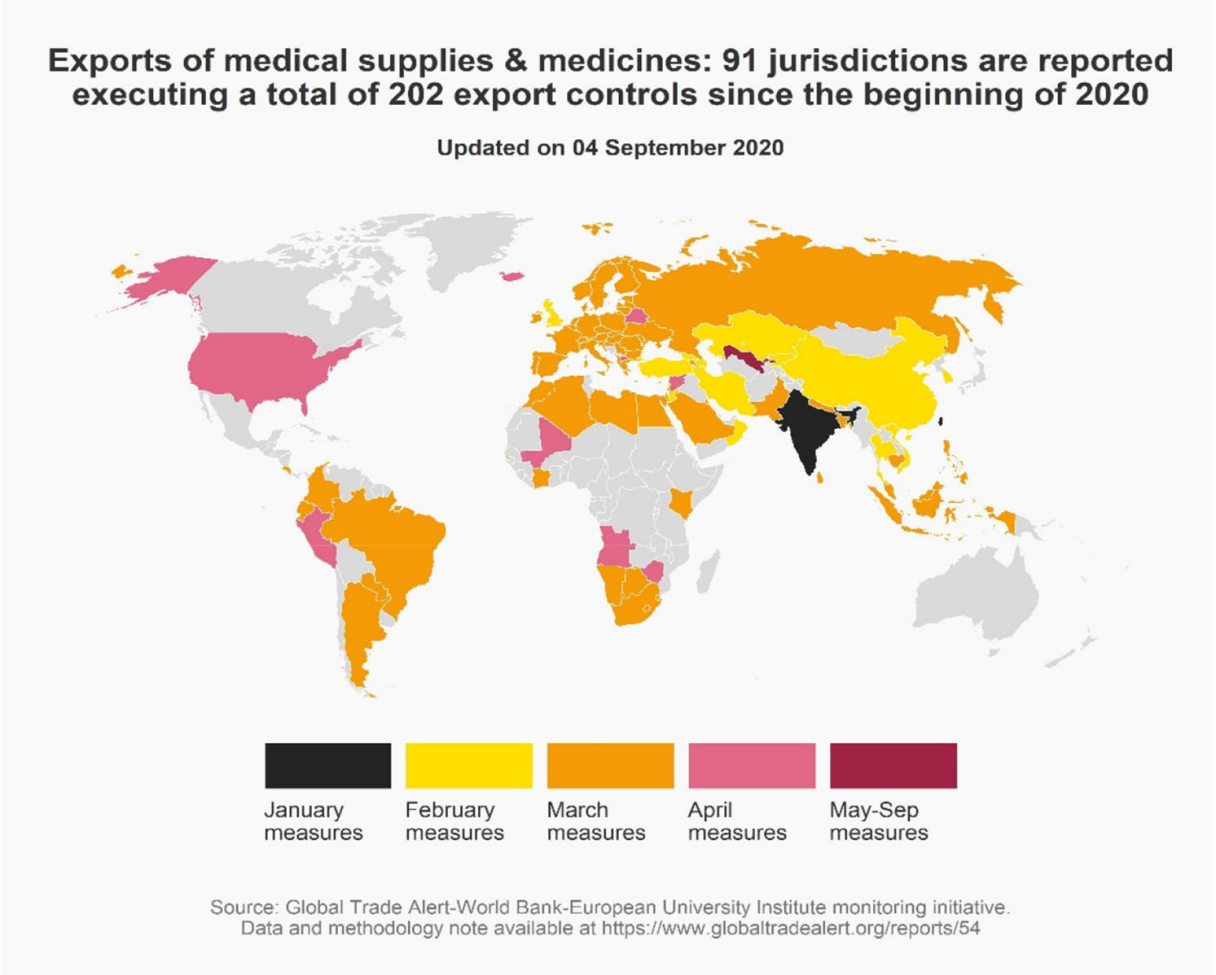
As the pandemic spread west, so did export controls on medical supplies and medicines.

The map in Fig. 1 shows the first month in which a trading nation introduced an export control on medical goods or medicines. Those export controls took many forms, including outright export bans, export authorization schemes, export quotas, non-automatic export license schemes, state requisition policies that *de facto* prevent or restrict exports, state exhortation to local producers not to ship to customers abroad, and requirements that local producers reserve a minimum percentage or amount of their production for the local market.^9^ All of these forms of export control were witnessed in the medicines and medical goods sector this year. A total of 91 jurisdictions executed 202 different types of export controls (for which implementation dates exist). March and April 2020 witnessed a frenzy of export controls being introduced. Notwithstanding the significant differences in complexity of the cross-border supply chains across medical goods and medicines, the impact of these export controls was to disrupt operations (see Forini, Hoekman, & Yildirim, 2020 for case study evidence).

While many national governments did resort to export curbs, there are notable exceptions. No export curbs on medical goods or medicines were introduced by Canada, Japan, Mexico, and New Zealand (or at least, none were detected). Australia introduced an export control that prevented buying personal protective equipment on the open market and shipping it abroad, however, no restriction was placed on the exports of these goods by Australian manufacturers. That the governments of these nations resisted the stampede towards export controls during one of the most serious global crises in recent times is telling, and may well influence corporate assessments of the political risk of such measures being introduced in the future, with potential implications for foreign direct investment and cross-border sourcing decisions.

**Figure 2:**
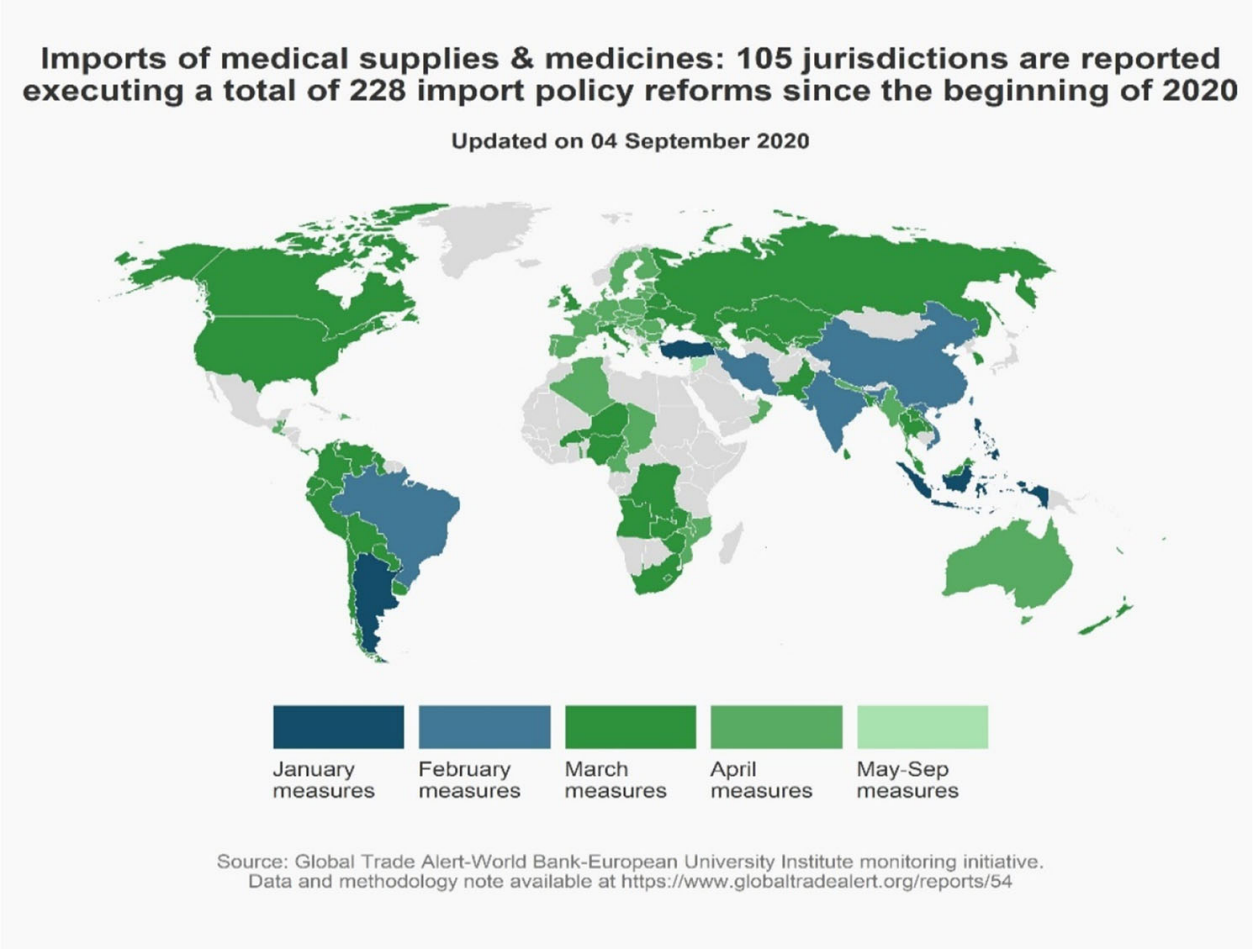
Over 100 nations cut import barriers on medical supplies and medicines since the pandemic began.

Exclusive focus on export controls, however, would miss the significant number of import-liberalizing measures undertaken in the medical goods and medicines sector since the beginning of 2020 (see Fig. 2). Before the pandemic hit, according to the WTO’s Tariff Download facility, 89 nations were charging tariffs on imported medical devices, 63 were doing so on imported medicines, 100 were taxing imported disinfectant, and 141 nations were taxing imports of soap (Evenett, 2020). One hundred and five jurisdictions took a total of 228 steps to ease imports of these products. Arguably, the contribution of cross-border supply chains in medical goods and medicines to fighting the pandemic was enhanced by the numerous trade reforms undertaken this year. Here, public policy complements commercial imperatives – unlike the case of export controls.

**Figure 3:**
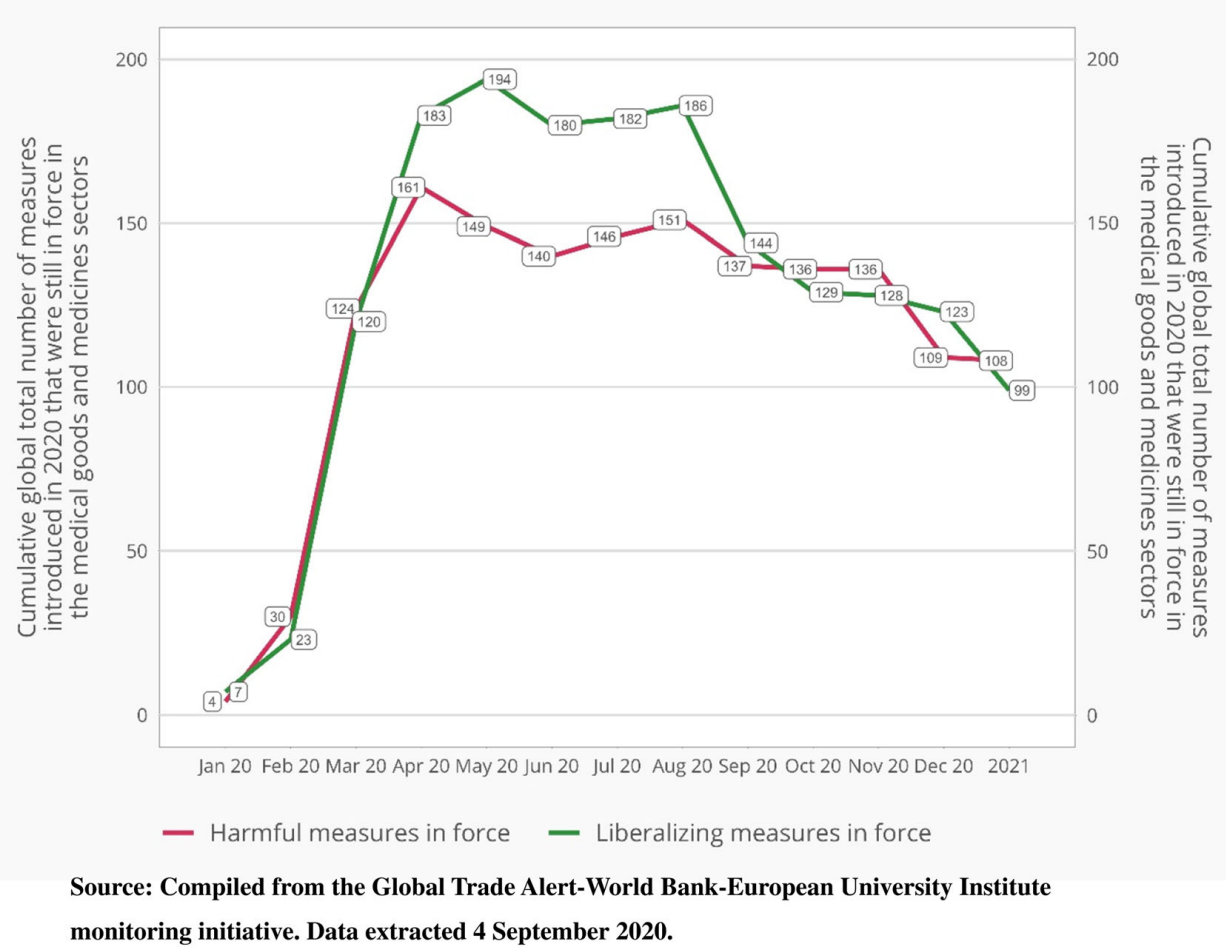
Around 100 of the import reforms and export controls on medical goods and medicines have no announced phase-out date. *Source*: Compiled from the Global Trade Alert-World Bank- European University Institute monitoring initiative. Data extracted September 4, 2020

The question arises as to whether these developments will likely result in a clear break with pre-pandemic trade policies in the medicines and medical goods sector. If the export curbs and import liberalization measures were temporary, then there may be doubts on this score, implying the pandemic might have little lasting impact on trade flows. Not only was information collected on when a measure came into force but also when it was scheduled to lapse. In Fig. 3, for each month this year and for 2021 (all months taken together), the total number of export controls and import reforms in effect is plotted. Two important findings emerge. First, approximately 100 export controls have no phase-out date – this is also the case for a comparable number of import reforms, suggesting that supply chains in this sector may need to be altered in light of a non-transitory change in the trade policy landscape facing firms.

Second, although the total number of import reforms introduced since the beginning of this year (228) exceeds the total number of new export controls (202), only during the months April 2020 to August 2020 did the total number of import reforms in effect clearly exceed the comparable totals for export controls. Of course, counts of measures introduced need not reflect the scale of commerce affected, still the evidence does not point to an unperturbed trading environment for cross-border supply chains in medical goods and medicines.

No discussion of developments in the medical goods and medicines sector would be complete without reference to surges in demand for these products that followed the global spread of COVID-19. In March 2020, the WHO stated “To meet rising global demand, WHO estimates that industry must increase manufacturing by 40 per cent” (WHO, 2020). In May 2020, the OECD went further, reporting back-of-the-envelope estimates that equipping Chinese medical, manufacturing, and transport workers with masks would require 240 million per day (OECD, 2020). The OECD branded this estimate “conservative” and noted that it exceeded the 20 million masks produced per day in China in January 2020. Overall, the OECD (2020) concluded “No country can meet the increased demand for face masks alone,” a conclusion that implies that cross-border supply would serve a useful societal purpose.

However, policies by major exporters of personal protective equipment (PPE) and other medical goods that *de jure* or *de facto* limit exports reduce supplies to the world market. As Bown (2020) points out, “As the coronavirus took hold in China in January and February 2020, there was a considerable increase in Chinese demand for PPE. The result was both more Chinese imports and fewer Chinese exports. This reduced China’s *net exports* of PPE, diminishing supplies available to the rest of the world.”^10^ A compounding factor was bottlenecks in domestic and international distribution arising during the pandemic.

Production of essential medical kits did ramp up in Q1 and Q2 of 2020. The Chinese State Council reported that in April production of N-95 masks and non-N95 masks had increased 38 and 34 times, respectively, over February production levels. Daily production of the latter masks reached the 200 million mark mentioned above in OECD (2020). Daily production of PPE was reported to have risen by April 2020 to 90 times the level seen in January 2020.^11^

### Food and Agri-food

**Figure 4:**
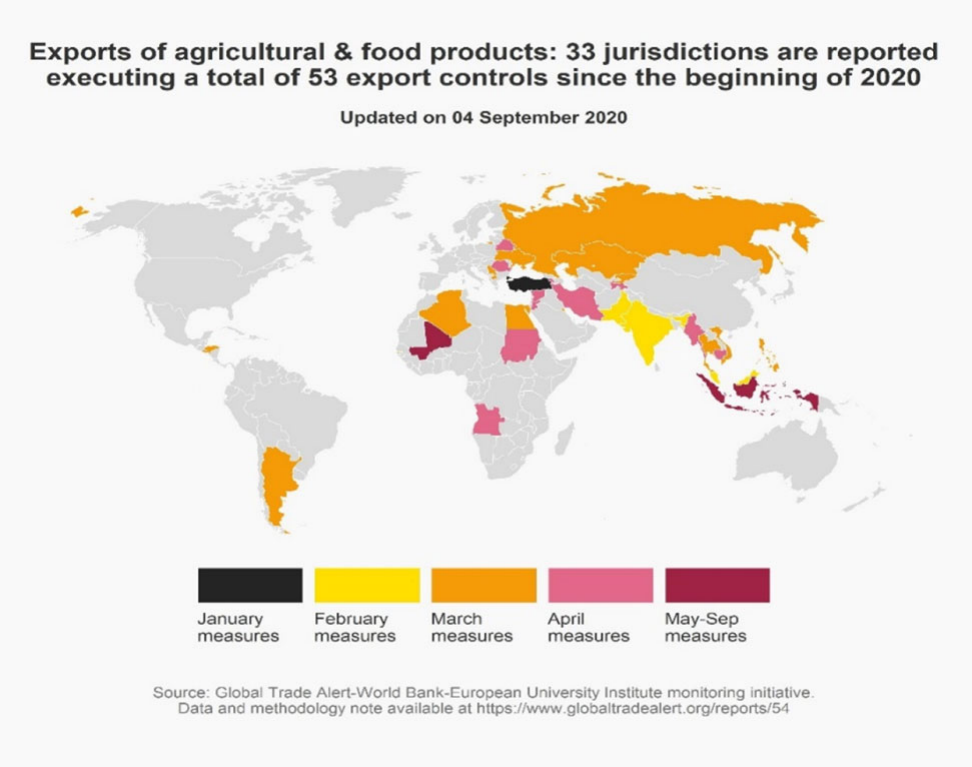
Far fewer export curbs in food were introduced than in medical supplies and medicines.

Fears that the COVID-19 pandemic would lead to near-term food shortages – which in turn would trigger export restrictions on food – did not come to pass (see Fig. 4). This contrasted with the sharp rise in the number of such export curbs in 2007–2008 when fears of food security were uppermost in many policymakers’ minds (Cullen, 2020). Thirty-three jurisdictions introduced a total of 53 export controls on food at some point during 2020. As Fig. 4 shows, unlike medicines and medical goods, the majority of those controls were not introduced in March and April 2020 but were spread more evenly across the first 9 months of 2020, suggesting a different dynamic was at work.

Of the major agricultural commodity exporters, Russia significantly tightened an export quota on grains in April 2020 and reversed course in July 2020. Vietnam, a major exporter of rice, introduced export curbs on March 25, 2020, that were reversed in steps thereafter. In the latter case, pressure from rice farmers was reported to be a decisive factor.

**Figure 5:**
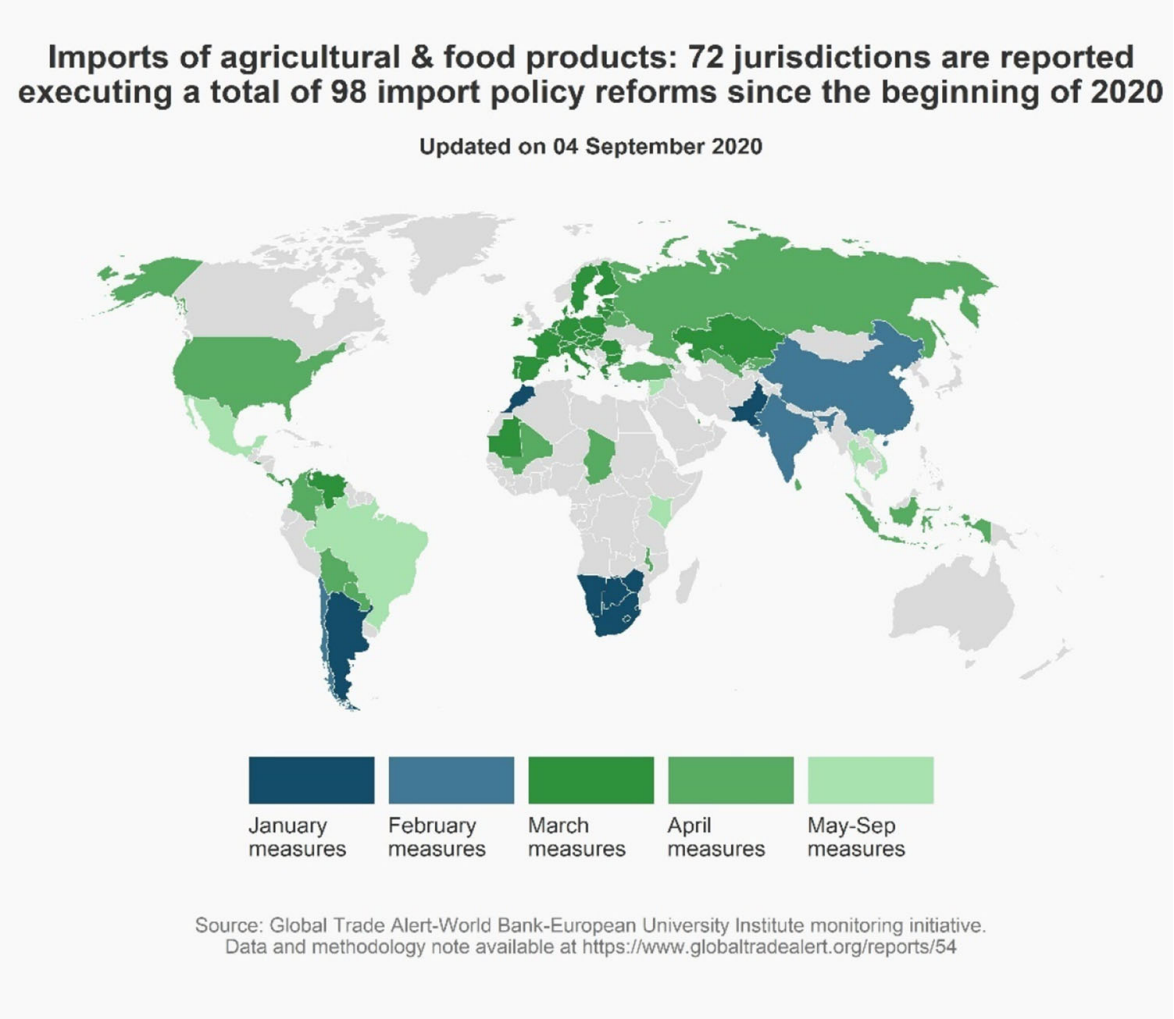
Many more import reforms were introduced than export curbs in the food sector, January–September 2020.

Both the total number of nations liberalizing cross-border trade in food and agri-commodities as well as the total instances of such reforms exceeded those for food export curbs (compare Figs. 4 and 5). Although several large emerging markets introduced food trade reforms in January and February 2020, just under half of the total number of reforms (47) were introduced in March and April 2020. Another 32 were introduced in the months that followed. A total of 62 of these import reforms have announced phase-out dates.

One argument that has been advanced as to why many more governments rushed to impose export curbs in medicines and medical goods as opposed to food relates to transparency (Bown, 2020). After the 2007–2008 food security scare, governments established the Agriculture Market Information System (AMIS), whose task is to provide accurate information on current food stocks and prices. This is said to have allayed fears this year about the availability of food.

No such global monitoring system exists for the medical goods and medicines sectors. While discussions of supply-chain transparency typically refer to the information a firm has about the upstream and downstream commercial counterparts, a global monitoring system for the medicines and medical goods sector would require a significantly higher degree of transparency, as governments would have access to this information, presumably for each cross-border supply chain of a certain scale.

## HAD GLOBALIZATION GONE TOO FAR? ASSESSING CLAIMS OF OVERDEPENDENCE ON CHINA

Once significant shortages arose in medical goods, a blame game ensued. Rather than acknowledge the role that surges in demand played or accepting any culpability for the export restrictions that they had imposed in creating shortages abroad, a remarkable number of senior policymakers blamed the configuration of pre-pandemic supply chains.

That many of those supply chains involved production in China where COVID-19 originated and, given the slump in Chinese exports of medical supplies in January and February, added a further twist. Several policymakers developed a broader critique, essentially that globalization had gone too far and created an overdependence on China that afforded that country too much leverage in times of crisis.

Add in the geo-political rivalry between China and the United States, in which relations were raw as a result of the ongoing trade war, and where the United States began demanding its allies take its side against China, then the critique of a prevalent form of international corporate organization – namely cross-border supply chains – acquired an even harder edge. Tellingly, this critique was not confined to those policymakers critical of offshoring before the pandemic struck.

The purpose of this section is to document the breadth of the shift in policymakers’ thinking and then critically evaluate that shift using a variety of evidence and expert judgement from regulators (and not from scholars or others that have advocated international economic integration).

### Statements by Policymakers, Relevant Context, and Supply Chain-Related Policy Intervention

This account starts in the United States. Economic nationalists in the Trump Administration were quick to seize on shortages in the medical goods and medicines sector. Dr. Peter Navarro, Assistant to the President and Director of the Office of Trade and Manufacturing Policy, stated at a White House press conference in the presence of President Trump:

One of the things this crisis has taught us, sir, is that we are dangerously over dependent on a global supply chain. Never again should we depend on the rest of the world for essential medicines and countermeasures.^12^

The United States representative drew broader lessons about the root causes of the shortages and future U.S. policy. Ambassador Robert E. Lighthizer told G20 trade ministers in March 2020:

Unfortunately, like others, we are learning in this crisis that over-dependence on other countries as a source of cheap medical products and supplies has created a strategic vulnerability to our economy… For the United States, we are encouraging diversification of supply chains and seeking to promote more manufacturing at home.^13^

This critique from Trump Administration officials comes on top of pre-pandemic concerns raised in the United States Congress about Chinese industrial policy and its implications for the health of the American public, amongst other concerns. For example, the U.S. Senate Committee on Small Business and Entrepreneurship, under chairmanship of U.S. Senator Marco Rubio, issued on February 12, 2019, a report about the *China 2025* industrial policy in which it was claimed:

The concentration of critical drug production in one country presents a threat to supply stability as well. For example, in 2016, a factory owned by the Chinese drug company Qilu exploded and triggered a global shortage of the drug piperacillin, an essential antibiotic for which the affected facility was the sole producer. In some cases, the Chinese government’s level of control over the supply chain already has resulted in direct leverage over trading partners (USC, 2019).

The apparent defense-related risks attendant to over-dependence on China had been singled out before the pandemic by U.S. Congressional representatives. For example, U.S. Senators Cotton and Warren in a letter^14^ to the U.S. Secretary of Defense dated December 5, 2019 argued:

An interruption in the supply of these products during an attack, either domestic or abroad, could have devastating consequences.

Specifically, overreliance on Chinese API exports raises the possibility that China could terminate or raise the cost of prescription drugs millions of Americans (including service members) rely on every day, in the event of escalating geopolitical tensions. This national security threat cannot be overstated. Should China seek to weaponize pharmaceuticals by restricting exports to the United States, incorporating lethal ingredients in final products, or any other means, our domestic pharmaceutical industry is not prepared to handle mass shortages for domestic or military use. Any interruption in the delivery of APIs or medicine would impact military readiness.

Such observations follow the publication in December 2017 of the Trump Administration’s first statement of its national security strategy.^15^ That document fused military, technological, and economic considerations and branded China (and Russia for that matter) a “revisionist power.”

Developments in the United States have been mirrored elsewhere, especially after the onset of the pandemic. Shinzo Abe, Prime Minister of Japan, the world’s third largest economy, went on record to declare the following shift in Japanese policy:

for those products with high added value and for which we are highly dependent on a single country, we intend to relocate the production bases to Japan. Regarding products that do not fall into this category, we aim to avoid relying on a single country and diversify production bases across a number of countries, including those of the Association of Southeast Asian Nations [ASEAN].^16^

Policymakers in the European Union hardened their position towards China, too. Even before the pandemic, in March 2019 the European Commission had branded China a “strategic competitor in the pursuit of technological leadership” and accused it of failing to open its markets on a reciprocal basis to European firms.^17^ That followed the French and German governments combining forces in February 2019 to launch *A Franco-German Manifesto for a European industrial policy fit for the 21st Century*.^18^ Although China is not referred to specifically in that document, which advocates greater resort to subsidization and a relaxation of EU merger review rules to facilitate the creation of regional champions amongst other initiatives, officials made no secret of the origin of the commercial threats they sought to address.

Developments in the medical goods and medicines sector were central to the case for a new approach to governing international commerce, and by implication international business. The French Minister of the Economy and Finance, Mr. Bruno Le Maire, has specifically advocated supply chain reform:

This pandemic is an occasion to reflect collectively on how to reorganise value chains; to reflect on the necessary investments for the health sector and on how to better protect our borders. And we shouldn’t be scared of the word “protection”. Protection is not the same as protectionism. Protection is the legitimate defense of our most strategic economic assets.^19^

The French President went further during a visit to French pharmaceutical manufacturer Sanofi in July 2020 observing that:

Everyone saw during this crisis that certain drugs were no longer manufactured in France or even in Europe. We must draw lessons from that...and the state is ready to invest in such reshoring projects.^20^

Mr. Le Maire’s German counterpart, Mr. Peter Altmaier, the Federal Minister for Economic Affairs and Energy, emphasized the importance of economic self-determination and the steps needed to attain it:

Minimizing one-sided dependencies in order to win back national sovereignty in sensitive areas is the right idea…I can well imagine a common European project for medicine production.^21^

If words alone determined the fate of cross-border supply chains, their days would be numbered for those that implicate China, at least in respect to essential goods such as medicines and medical goods. But have governments backed up these statements with policy initiatives? Here the evidence is mixed.

Perhaps first off the mark was Japan, whose stimulus plan announced on April 7, 2020 included 220 billion Yen (approximately $2 billion) in financial grants for firms moving production facilities out of China.^22^ In July 2020, Japan announced that 87 firms had successfully applied for $653 million of financial support to do so. Another 30 companies will receive financial support to move production facilities to the ASEAN region.

The United States has deployed the Defense Production Act of 1950 to, amongst others, offer financial incentives to expand production within the United States.^23^ At the direction of the president, the U.S. International Development Finance Corporation signed on July 28, 2020 a letter of intent with Kodak to commence production of pharmaceuticals in the United States.^24^ Kodak was to be given a state loan of $765 million to do so. Moreover, the U.S. Departments of Defense and Health and Human Services will “invest nearly $630 million to expand the domestic industrial base for medical resource suppliers,” according to a U.S. Department of Defense press release of August21, 2020.^25^ Unlike the Japanese approach of offering carrots to firms to move out of China, presidential rhetoric aside, during the pandemic the U.S. has offered financial incentives to expand production at home, thereby substituting imports.

In contrast, the French and German stimulus packages announced in the third quarter of 2020 do not appear to earmark specific funds for repatriating supply chains, somewhat undercutting the statement reported above by President Macron. Meanwhile, Canada,^26^ Brazil,^27^ India,^28^ Italy,^29^ Japan,^30^ Korea^31^, and Russia^32^ have provided state aid to producers of medical supplies and medicines in the first 8 months of 2020.

The Chinese government appears to be having second thoughts about the degree to which exports should contribute towards national economic growth. In May 2020, President Xi announced a new “dual circulation” initiative motivated in part, it was reported, by rising protectionism abroad.^33^ According to one well-placed observer, this

new economic strategy calls for the country to continue to expand domestic production for exports (“international circulation”) while shifting the economy towards greater relative emphasis on production for domestic consumption (“internal circulation)” (Pettis, 2020).^34^

At the time of this writing, few attendant policy interventions have been made public. A State Council announcement on June 17, 2020 indicated that different forms of financial support would be made available to selected firms that shift sales from export to domestic markets.^35^ Meanwhile, a subsequent State Council announcement on August 5, 2020 offered larger incentives to foreign firms investing in China.^36^ It would seem, therefore, that Chinese policymakers are altering the desired mix of contributions from domestic and international business to their nation’s economic development.

Overall, some governments have backed up their rhetoric on supply chain reconfiguration with financial support. Whether that support is sustained, augmented, or indeed is enough to incentivize many firms to reconfigure their supply chains is too soon to say. Nevertheless, if this does come to pass, there were plenty of warnings of what was to come delivered by policymakers during the fraught early months of the pandemic.

### Global Flows in PPE

Having documented what could become a significant shift in the incentives and constraints facing international business, the discussion now turns to whether the premise of the many senior policymakers’ critiques can be sustained empirically. The principal contention examined here is that, for whatever reason, before the pandemic, the rest of the world grew too dependent on China for essential goods. Given the salience of PPE supplies during the early months of the pandemic, the latest available pre-pandemic international trade data are used to shed light on the sourcing patterns for masks and the like.

The policy discussion on over-dependence has a quality similar to U.S. Supreme Court Justice Potter Stewart’s definition of obscenity: “I know it when I see it.” An economic approach to the problem of the over-dependence may add some coherence to this discussion, even if it does not provide a specific test. A nation is more likely to be over-dependent on a trading partner to supply a product when the latter’s share of total imports is higher and the number of credible alternative supplies is lower.^37^ In this section, we rely on the most fine-grained international global trade data available for 2015 to 2018 to identify which nations are very dependent on China for supplies of PPE.

**Figure 6:**
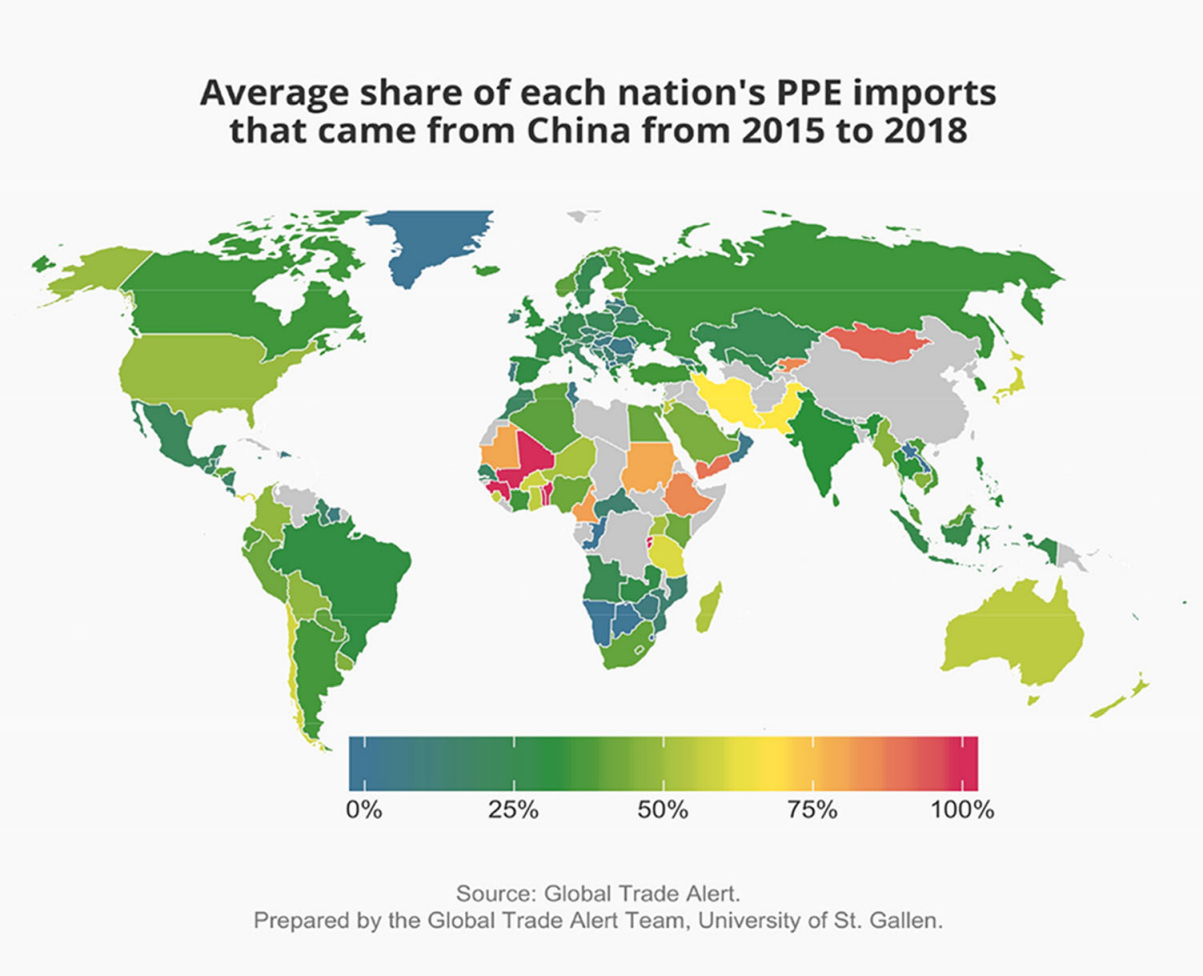
Before the pandemic struck, few nations sourced more than half of their PPE imports from China.

Figure 6 reports the average shares of total imports of PPE that each nation sourced from China during the 4 years 2015 to 2018. A 4-year average is less likely to be distorted by errant trade flows for a single year. Care is needed in interpreting the findings in this figure for the average Chinese import share is not equal to the average Chinese share in domestic consumption. Indeed, the larger is the amount of domestic production of PPE, the smaller is the country’s reliance on imports for the supply of PPE. Consequently, the Chinese import share provides an upper bound on the dependence of a nation’s PPE consumption on China.

The map in Fig. 6 reveals that outside of Africa, only a few nations source over 75% of their PPE from China. No American nations (north or south), no European nation, and no member of the Commonwealth of Independent States (which includes Russia) sources more than half of their PPE imports from China. Of the Group of G20 nations, China accounts for moderate to high shares of PPE imports by Australia and Japan. As will soon become clear, Japan is itself a major exporter of PPE, so that leaves Australia as being potentially vulnerable to arbitrary changes in Chinese supplies of PPE.

The second dimension to over-dependence is the availability of alternative suppliers. Using global trade data, it is possible to identify which nations supplied between $500 million and $1 billion and more than $1 billion of PPE exports to the world market before the pandemic struck. The fewer such suppliers, the graver the concerns that there is a Chinese “chokepoint” in the supply of PPE.

**Figure 7:**
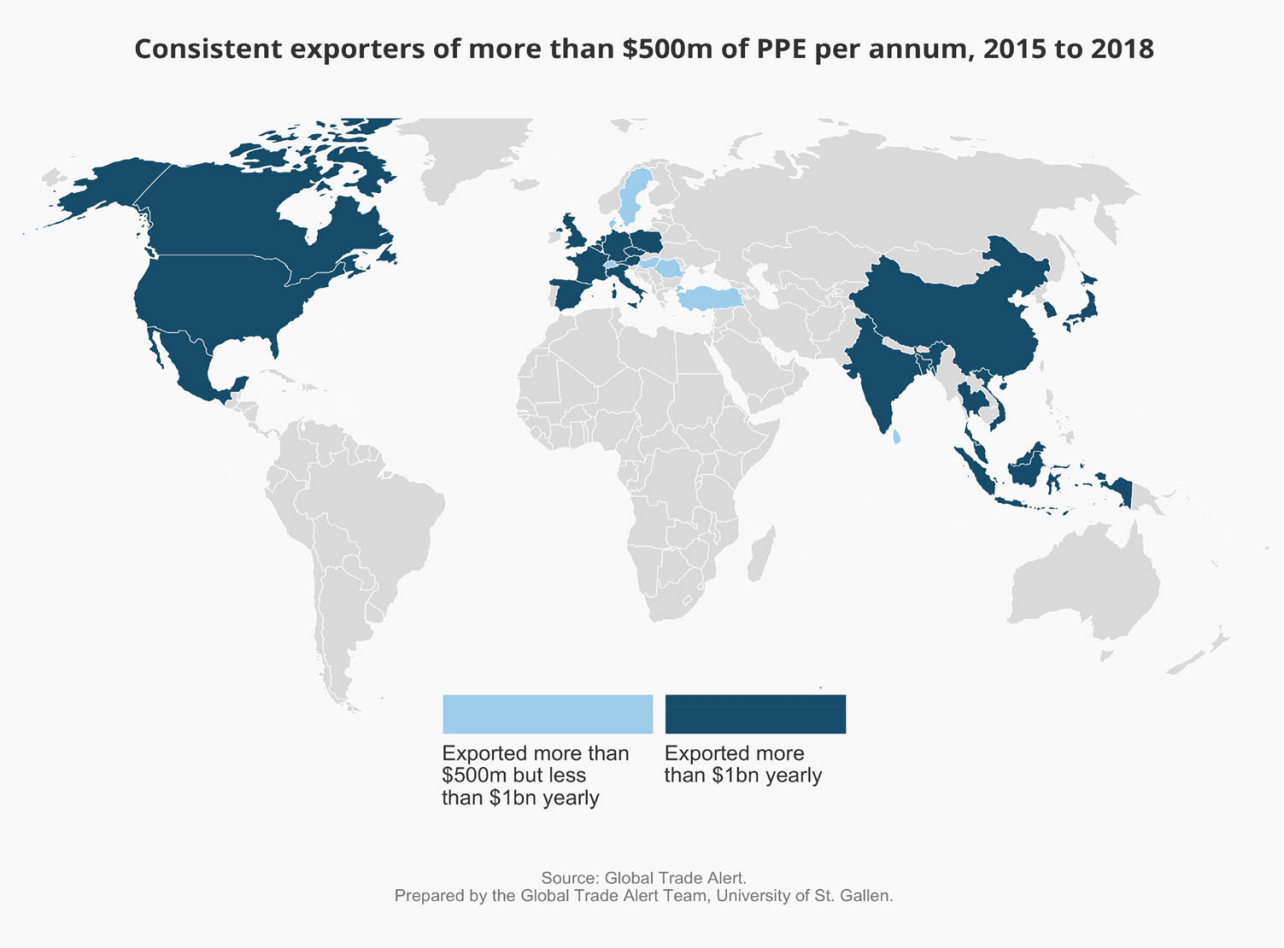
Before the pandemic, many nations consistently exported more than $500 million of PPE per annum.

Figure 7 produces a map showing which nations consistently export more than half a billion U.S. dollars of PPE. The headline finding is that buyers of PPE have many nations to turn to if China were to cut off or restrict supplies. Moreover, those alternative suppliers are spread across East Asia, North America, and Western Europe (not to mention Turkey). Therefore, even if a government found itself in a stand-off with neighboring countries, it could still source PPE from other regions.

**Figure 8:**
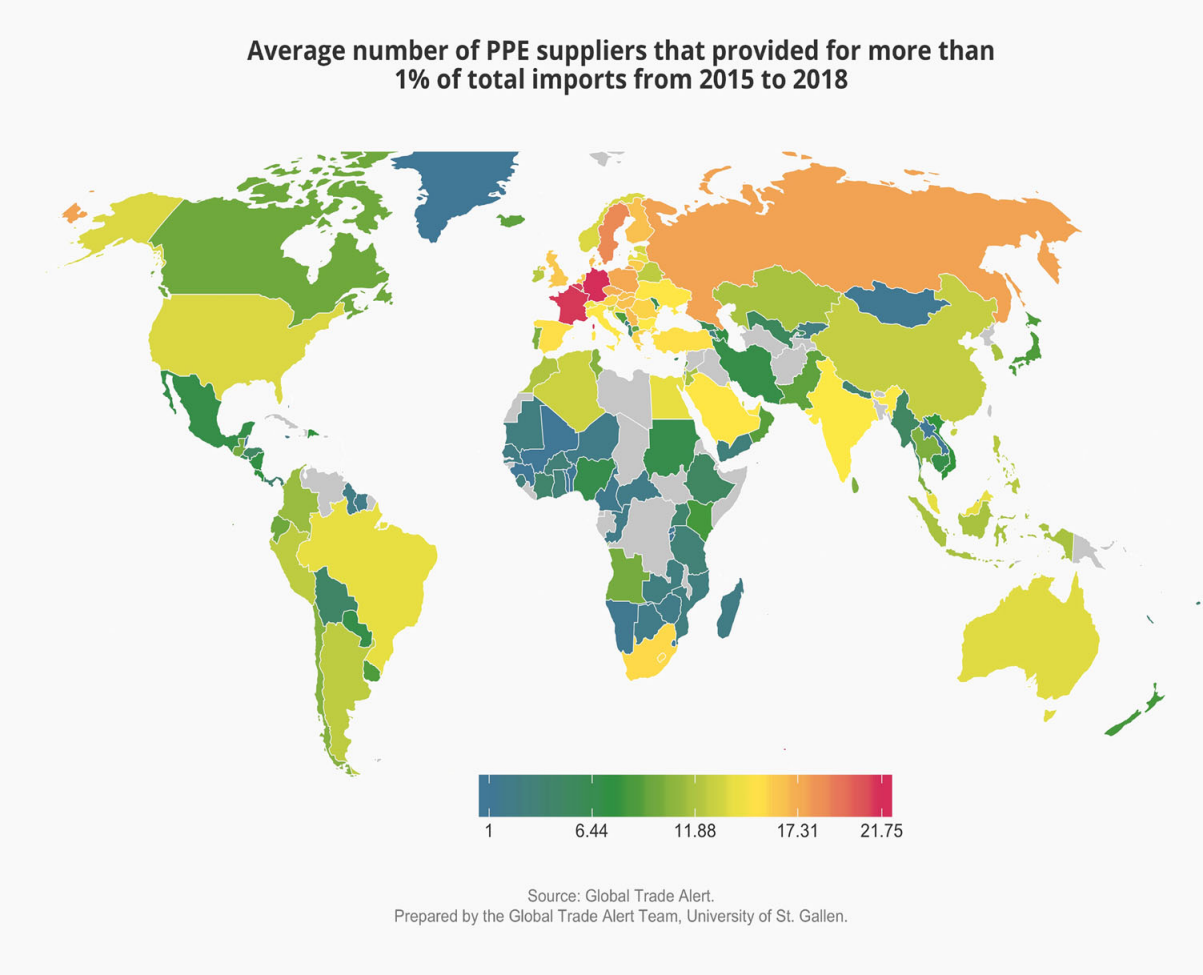
Outside of Africa, remarkably few nations had concentrated sourcing patterns for PPE before the pandemic.

Did the existence of so many alternative suppliers before the pandemic translate into diversified sourcing patterns from a wide range of PPE exporters? Figure 8 answers that question by showing for each importing nation the number of foreign trading partners that furnish more than 1% of its total imports. Again, Mongolia and certain African nations (along with Greenland) stand out as concentrating their imports of PPE in a small number of foreign suppliers.

None of the members of the G-20 economies have fewer than six suppliers supplying more than 1% of their imported needs (recall these needs can also be met by domestic PPE production). France and Germany have an unusually large number of foreign suppliers that deliver more than 1% of their import bills for PPE, undercutting claims by these nations’ policymakers that before the pandemic they were too dependent on any one supplier.

Taken together, these findings based on the latest available fine-grained global trade data for personal protective equipment call into question that cross-border supply chains resulted in undiversified sourcing patterns. In fact, a large number of nations consistently exported PPE and, whether by accident or design, more nations availed themselves of this bounty and diversified sourcing patterns were the result.

### Evidence from Detailed Import Data

In advancing the over-dependence thesis, many advocates refer to the sourcing patterns of specific medical goods or medicines. This statement by Pletka and Scissors (2020) is typical of this line of argument:

Consider that Chinese firms are said to supply more than 90% of US antibiotics, 70% of acetaminophen (that’s Tylenol), and almost half of the anti-coagulant heparin.

Other examples can be found in Rosemary Gibson’s testimony to the U.S.–China Economic and Security Review Commission on July 31, 2019 in which she asserted that Chinese producers of penicillin formed a cartel and drove European and U.S. producers out of the market (Gibson, 2019). She also contends:

…China’s vitamin C (ascorbic acid) cartel forced the closure of the last U.S. production facility, and the last aspirin (acetylsalicylic acid) manufacturing facility ceased business because of predatory pricing by Chinese firms. Baxter Healthcare switched heparin suppliers from Wisconsin to China, and a lethal contaminant in heparin was later found that killed hundreds of Americans (Gibson, 2019).

The central research question is whether anecdotes like these are representative. Fortunately, the European Union and the United States collect very detailed import data, far more detailed than that made available by the United Nations and used in the sub-section directly above.

With respect to the European Union, it was possible to identify 154 product categories at the eight-digit level of disaggregation that correspond to medical goods and medicines.^38^ For these product categories, it was possible to identify the number of instances where France, Germany, or the United Kingdom imported more than half of those products from a single trading partner. This provides some indication of the degree to which international sourcing patterns are concentrated. Furthermore, once those instances are identified, it is possible to identify the trading partners responsible for those shipments. Table 1 summarizes the findings.

**Table 1:** Before the pandemic, China was the majority supplier of only a small number of medical goods and medicines to France, Germany, and the United Kingdom

Importer	Number of medical goods and medicine categories where largest foreign supplier accounts for more than half total imports (maximum 154 product lines)	Number of medical goods and medicine categories where more than half of imports are from China (maximum 154 product lines)	Total value of imports (USD in millions) where China is majority foreign supplier
France	48	4 (USA = 8, Germany = 16)	31
Germany	35	6 (USA = 7)	152
United Kingdom	57	6 (Germany = 12, USA = 17)	168

*Source*: Computed from fine-grained (eight-digit) product annual data for 2019 available from Eurostat.

In 57 out of the 154 products (or 37% of cases), the United Kingdom sourced more than half of their imports from a single country in the year before the pandemic (2019). The comparable percentages for France and Germany are lower, 31% and 23% respectively. On the face of it, this might suggest that concentrated sourcing is a concern in a range of imported medical goods and medicines. However, as column 3 of Table 1 shows, in no more than six products was the majority foreign supplier Chinese.

France, whose officials have made so much of the over-dependence thesis, saw just four medical and medicine products where China was the majority supplier. If anything, according to the statistics reported in the third column of Table 1, for every imported medical good that China is the majority foreign supplier there are two for which this is the case from the United States and four products where Germany is the majority foreign supplier. Such summary statistics put France’s apparent over-dependence on Chinese imported medical goods and medicines in context. The last column of Table 1 reveals the small values of total imports where China was the majority supplier of imported medicines and medical goods to these leading European economies before the pandemic.

In the case of the United States, the most fine-grained import data available are at the ten-digit level of disaggregation, available from the U.S. International Trade Commission. A total of 326 product categories relating to imported medical supplies, medical equipment, PPE, and medicines were identified and import data for 2019 extracted. Summary statistics on trading partners exporting more than $1 billion of these goods to the United States in 2019 are presented in Table 2 for each of the four product groups mentioned in the last sentence.

**Table 2:** China is the largest foreign supplier to the U.S. in less than 30% of medical goods and medicine product categories

Trading partner	US medical imports in 2019 from trading partner, $bn	CAGR of US medical imports from trading partner 2017–2019, %	Medicines (pharmaceuticals) (73 ten-digit product codes)		Medical supplies (83 ten-digit product codes)		Medical equipment (75 ten-digit product codes)		Personal protective products (99 ten-digit product codes)		Import share in products where trading partner is largest foreign supplier to the US
			% trading partners shipments to the US by value	Number of times the trading partner is the largest supplier to USA	% trading partners shipments to the US by value	Number of times the trading partner is the largest supplier to USA	% trading partners shipments to the US by value	Number of times the trading partner is the largest supplier to USA	% trading partners shipments to the US by value	Number of times the trading partner is the largest supplier to USA	
Ireland	29.462	4.96	80.53	8	11.59	2	7.77	3	0.10	0	50.51
Germany	23.636	16.43	61.05	6	14.44	6	20.51	**19**	4.01	10	37.07
Switzerland	17.317	14.04	81.52	3	10.12	1	7.80	3	0.55	0	36.78
China	14.249	**5.47**	5.06	3	19.42	**22**	21.17	16	54.34	**54**	**51.21**
Mexico	11.001	8.30	3.05	0	27.81	7	48.92	12	20.22	7	35.76
Italy	8.945	26.76	91.84	5	2.77	1	3.12	2	2.28	1	69.86
Canada	8.696	13.03	57.84	11	15.67	8	8.36	3	18.12	6	50.71
India	8.060	11.87	92.50	**17**	1.48	3	3.63	0	2.39	0	28.66
Japan	7.865	17.40	52.33	1	13.23	4	25.06	2	9.39	7	54.56
UK	6.866	5.51	62.86	7	22.17	2	9.71	2	5.26	1	25.88
Denmark	6.838	37.41	90.34	1	7.48	2	1.50	0	0.67	1	53.06
Belgium	6.572	89.33	93.84	3	4.05	2	0.82	0	1.30	0	50.18
Singapore	5.923	13.75	62.23	1	12.40	1	23.62	3	1.75	1	35.22
France	4.784	9.21	65.90	2	14.02	1	12.59	0	7.50	2	33.02
Israel	3.518	− 18.65	55.58	1	13.16	1	24.52	2	6.74	2	34.47
South Korea	2.972	15.60	70.24	0	8.01	1	12.74	2	9.02	4	34.54
Malaysia	2.470	13.36	0.07	1	73.20	5	23.54	1	3.19	0	63.26
Australia	1.572	16.85	14.17	0	64.82	1	18.47	0	2.55	0	34.81
Thailand	1.279	16.01	0.73	0	62.94	1	17.36	1	18.97	0	55.00
Taiwan	1.262	12.08	15.27	0	16.32	1	27.89	0	40.52	0	19.17

*Note*: Compiled from U.S. import data for 330 product lines at the ten-digit level of disaggregation of the US Harmonized Trading System.

*Note*: Data highlighted in bold represent the largest number reported in a particular column.

With respect to medicines imported into the United States, India is the largest supplier in 17 of the 73 product categories. China is the largest foreign supplier in just three cases. With respect to medical equipment, Germany is the largest foreign supplier in 19 out of 75 product lines. China comes second here, being the largest foreign supplier in 16 products. With respect to medical supplies, China is the largest foreign supplier most often, 22 times out of a total of 83 product lines. Where China stands out as the largest foreign supplier is in PPE, where in 54 of 99 products it ships the most to the United States.

Counts are useful but ought to be supplemented by some measure of the scale of trade implicated. This is where the final column of Table 2 comes in as it reports, for the products where a trading partner is the largest supplier, the percentage of total imports into the United States that come from the trading partner in question. In China’s case, in the 95 cases where it was the largest foreign supplier before the pandemic, its share of imports was 52%. Add in the fact that U.S. domestic production of these goods can be used to supply American buyers, then the share of the U.S. market supplied from China almost certainly falls below 50%.^39^

What do these findings imply about the U.S. foreign sourcing patterns for these products before the pandemic? At most, U.S. dependence on China as a source is largely found in PPE and, even there, there are 45 PPE products where China is not the largest foreign supplier. For the 45% of Chinese medical goods and medicine exports that are not PPE, in just over a sixth of cases (17.7%) was China the largest supplier.

Moreover, contrary to any suggestions that China’s exports of medical goods and medicines were surging before the pandemic and knocking out other foreign suppliers, in fact the cumulative average growth rate of such imports from 2017 to 2019 was under 6%, well below the growth rate witnessed by many other U.S. trading partners. Concentration of U.S. imports of medicines and medical goods on China is at best a localized problem. Claims that there was a generalized over-dependence on China can be rejected. The anecdotes deployed by advocates of the over-dependence thesis are not representative of the broader trends in U.S. foreign sourcing behavior of medical goods and medicines.

### Statements and Analysis by the U.S. Food and Drug Administration

The findings based on detailed import data presented above are confirmed by the statements and analysis of U.S. officials associated with the Food and Drug Administration (FDA). In a Fox News television interview on April 5, 2020, at a time when many governments were imposing export curbs on medical goods and medicines, the Commissioner of the FDA, Dr. Stephen Hahn, made the following remarks in response to questions put to him. On the subject of shortages he observed:

I can tell the American people that critical medications are available, but there are spot shortages because of increased demand, so we are working very closely with domestic and international suppliers to increase the supply of those.

On the subject of suppliers, including foreign suppliers using leverage:

Right now, we don’t have any evidence that there’s a drug in short supply because of anyone blocking the active pharmaceutical agreement ingredients coming to us.

Looking forward, he argued:

We absolutely must address the issue of redundancy in our manufacturing, and we must absolutely make an effort to have domestic manufacturing as well.

Comments such as these follow a long line of statements by FDA officials about what they do and do not know about U.S. over-dependence on foreign suppliers and on shortages. For example, on October 29, 2019, in testimony^40^ before the U.S. House Committee on Energy and Commerce’s Subcommittee on Health, Dr. Janet Woodcock, Director of the Center for Drug Evaluation and Research, summarized the national security findings of her analysis of over-dependence on China as follows:

The FDA’s information shows that, overall, the number of China’s API facilities is somewhat smaller than the United States, but comparable in size and growing. However, because of the limitations of available data, we cannot assess the extent of U.S. dependence on China. For instance, we do not have information about the volume of API being produced in China or even in the United States, or how much of China’s API output reaches the U.S. market through other countries.

Similarly, we do not have information that would enable us to assess the resilience of the U.S. manufacturing base, should it be tested by China’s withdrawal from supplying the U.S. market. We do know that the U.S. drug supply is being compromised by drug shortages, in most cases triggered by manufacturing quality problems by U.S.-based as well as foreign producers.

Such comments are consistent with the observations made about the lack of transparency in cross-border supply chains in medical goods and medicines in the last section of this paper. They also imply that the data were not available to conclude, as some policymakers and analysts have done, that before the pandemic dependence on China was a national security threat to the United States.

Since the pandemic was declared, the FDA has given updates on the availability of medicines. On February 27, 2020, its Commissioner shed light on the current supply of medicines, including active steps it was taking to source from China^41^:

Since January 24, the FDA has been in touch with more than 180 manufacturers of human drugs, not only to remind them of applicable legal requirements for notifying the FDA of any anticipated supply disruptions, but also asking them to evaluate their entire supply chain, including active pharmaceutical ingredients (the main ingredient in the drug and part that produces the intended effects, e.g., acetaminophen) and other components manufactured in China.

Also, as part of our efforts, the FDA has identified about 20 other drugs, which solely source their active pharmaceutical ingredients or finished drug products from China. We have been in contact with those firms to assess whether they face any drug shortage risks due to the outbreak. None of these firms has reported any shortage to date. Also, these drugs are considered non-critical drugs.

As to the supply of medical equipment, Commissioner Hahn observed:

We are aware of 63 manufacturers which represent 72 facilities in China that produce essential medical devices; we have contacted all of them. Essential devices are those that may be prone to potential shortage if there is a supply disruption. We are aware that several of these facilities in China are adversely affected by COVID-19, citing workforce challenges, including the necessary quarantine of workers. While the FDA continues to assess whether manufacturing disruptions will affect overall market availability of these products, there are currently no reported shortages for these types of medical devices within the U.S. market.

Regarding personal protective equipment – surgical gowns, gloves, masks, respirator protective devices, or other medical equipment designed to protect the wearer from injury or the spread of infection or illness – the FDA has heard reports of increased market demand and supply challenges for some of these products. However, the FDA is currently not aware of specific widespread shortages of medical devices, but we are aware of reports from CDC and other U.S. partners of increased ordering of a range of human medical products through distributors as some healthcare facilities in the U.S. are preparing for potential needs if the outbreak becomes severe.

By March 2, 2020, the FDA noted^42^ that shortages were not pervasive but could become so:

Of note, the agencies are not currently aware of specific widespread shortages of personal protective equipment, but there are reports of increased ordering of these products and shortages have been observed in some U.S. health care institutions. The FDA and CDC are aware that as the COVID-19 outbreak continues to expand globally, the supply chain for these devices will continue to be substantially stressed as demand exceeds available supplies. Under the circumstances of this emergency, nationwide shortages are anticipated.

On March 28, 2020, the FDA acknowledged^43^ that there were shortages in PPE and ventilators but not in medicines. Foreign sourcing was seen as part of the solution, not the problem:

We are also open to importing PPE and other devices…The agency is taking steps to facilitate importation of PPE into the U.S. and we are ready and available to engage with importers to minimize disruptions during the importing process.

Indeed, the FDA claimed to be in contact with many manufacturers worldwide to meet surging demand:

The FDA has reached out to more than 1000 device manufacturing sites worldwide, focusing on essential devices. The outreach thus far has focused on two main types of essential devices: those that are in high demand due to the pandemic outbreak, such as PPE and ventilators, and devices that may be prone to potential shortage if there is a supply disruption.

On the basis of these comments, once demand for PPE and the like surged in the United States the FDA appeared willing to increase its “dependence” on foreign suppliers.

The FDA also monitors medicines shortages, producing both reports and data on them. Such information can be used to assess whether foreign suppliers, including Chinese suppliers, regularly cut off shipments to the U.S. market. In fact, on October 29, 2019, an FDA-led task force of U.S. Federal officials published a report on drug shortages as they refer to them. The following statement addresses both the consequences and, more importantly, the root causes of such shortages.

The Task Force found that the number of ongoing drug shortages has been rising, and that their impact is likely underappreciated. The Task Force analyzed 163 drugs that went into shortage from 2013 to 2017 and compared these medicines to similar drugs that did not go into shortage. Shortage drugs were more likely to be relatively low-price and financially unattractive drugs and were more likely to be sterile injectables. Shortages often occurred as a result of disruption in supply due to a variety of factors. Importantly, prices rarely rose after shortages began, and during shortages, production typically did not increase enough to restore supply to pre-shortage levels. Many manufacturers reported discontinuing the production of drugs before a shortage for commercial reasons (e.g., loss of profitability). These results suggest a broken marketplace, where scarcity of drugs in shortage or at risk for shortage does not result in the price increases predicted by basic economic principles. While there are no easy solutions to the problems identified, and there is no single cause of drug shortages, the Task Force offers three key recommendations to address the root causes of shortages.

Given the potential scale of impacts from drug shortages, and the fact that these impacts have continually been underestimated, it is likely that drug shortages will continue to persist absent major changes to this marketplace. The root causes of shortages involve economic factors that are driven by both private- and public-sector decision-making. This means that the types of enduring solutions proposed in the report will require multi-stakeholder efforts and rethinking business practices throughout all sectors of the health care system. It will also require a fuller characterization of the true costs of shortages and more comprehensive and reliable analysis of the effects shortages have on patients and the health care system (FDA 2019).

What is significant about these findings is that the root causes of medicine shortages are not blamed on cross-border supply chains but are much more complex in nature (Gereffi 2020). It is noteworthy that repatriation of production to the United States was not mentioned as a solution. The report also notes that Chinese and Indian firms are barred from supplying active pharmaceutical ingredients (API) to the U.S. Department of Defense, an observation that further casts doubt on the direct risk to the U.S. military of any Chinese attempt to limit shipments to the United States.

The FDA also maintains an up-to-date register of medicines shortages. For some entries, the FDA lists a reason or reasons for the shortage. For those shortages notified during 2020, information was extracted on the reasons provided by the FDA and this is summarized in Table 3. Of the 281 cases where reasons were given, 168 refer to demand increases. In only 26 cases were shortages of ingredients referred to. Even if all of those ingredient shortages were the responsibility of foreign suppliers, they would account for only 9% of the medicine shortages found this year by the FDA for which the cause could be identified. These statistics imply that surging demand rather than denial of supply were the predominant causes of medicine shortages in the United States this year.

**Table 3:** Reasons provided by the FDA for drug shortages. September 2020

Reason(s) given (listed in alphabetical order)	Number of times this reason was given
API shortage	6
Delay in shipping of the drug	10
Demand increase due to Covid-19	3
Demand increase for the drug	150
Demand increase for the drug and shortage of an active ingredient	4
Discontinuation of the manufacture of the drug	1
Limited API availability	2
Other	75
Regulatory delay	1
Requirements related to complying with good manufacturing practices and demand increase for the drug	11
Shortage of an active ingredient	17
Shortage of an inactive ingredient	1
*Subtotals:*	
All mentions of demand increases	168
All mentions of ingredient shortages	26

*Note*: This table refers to the 281 drug shortages that the FDA identified a reason for a drug shortage where the initial posting of the shortage occurred on or after January 1, 2020.

*Source*: https://www.accessdata.fda.gov/scripts/drugshortages/.

Overall, the picture that emerges about supply chains in medicines and medical goods from the health experts at the U.S. Food and Drug Administration is at odds with the critics of supply chains. The FDA acknowledged that supply chains came under strain during the pandemic and this was because of surging demand could not be met in the near term by ramping up supply, neither at home nor abroad. It is this supply and demand mismatch which is at the core of the problem, a finding reinforced by the FDA data on drug shortages this year in the U.S. market.

## CONCLUDING REMARKS: CUI BONO?

That nations can trade reduces the risk that they are tied to local firms for supplies. Greater choice, lower prices, and flexibility in sourcing were supposed to be distinct advantages of an open trading system. The build-up and evolution of supply chains over recent decades were a key building block, and much research has been devoted to this corporate form, the challenges it faces, and its developmental, economic, and societal impact.

The COVID-19 pandemic could have been the moment when firms operating cross-supply chains meaningfully contributed to tackling a major societal threat. That countries witnessed surges in infection at different times implies that smoothly functioning supply chains could ramp up production and ship medical supplies and medicines to destinations where demand was surging. No such luck.

Instead, senior policymakers in many of the world’s leading economies, and not just those from governments associated with populist policies and economic nationalism, have drawn negative conclusions about this prominent corporate organizational form. Not only that, many policymakers have made statements consistent with the proposition that globalization had gone too far before the pandemic. Numerous governments have taken steps to encourage the repatriation of production or to stimulate domestic production to displace imports.

Analysts can respond to these statements by policymakers in at least four ways. First, some might aver “talk is cheap.” This may not be the appropriate conclusion, as governments have begun backing up their critique with policy intervention. Time will tell if these interventions are sustained.

Second, some might dismiss these statements as blame shifting. Given that it was often the same policymakers that disrupted supply chains in the medical goods and medicines sector once the coronavirus spread by resorting to over 200 export controls, there may be something to this. The wrinkle with this argument is that the Japanese government, which did not impose any export bans, has also joined the critique of cross-border supply chains and is financially supporting Japanese firms that move production facilities out of China.

Third, analysts may decide to critically evaluate the policymakers’ critique. That was the purpose of a large part of this paper and it should be evident that, by any reasonable standard of logic and evidence, the case made against cross-border supply chains is unconvincing. No objective standard by which cross-border supply chains were to be judged was enunciated by policymakers, although thinking through what such a standard should be in the context of a pandemic is worthwhile. In extremis, how should cross-border supply chains be judged?

A fourth reaction of analysts to the apparent shift in policymakers’ attitudes towards cross-border supply chains might be to ask “what’s really going on here?” It was not the purpose of this paper to address this question. Future research could generate powerful insights into the factors that influence when and how policymakers gauge the performance of international business. Potentially important pieces of the puzzle were, however, presented here. These include the pandemic’s attendant demand surge and high-profile media reports of shortages which likely reflect the limited incentives that firms have to maintain excess production capacity during normal times (see also Gereffi 2020).^44^ That few stockpiles were maintained by the public or private sectors in many countries may be another element.

But surely the changing geopolitical context must be considered as well. There are now important business, national security, non-governmental, and religious constituencies in the largest economies of the world that are alarmed by China’s rise for a variety of reasons. Did the pandemic create the opportunity to traduce cross-border supply chains with an eye to redrawing the terms upon which international business is enjoined to operate? Who benefits from this game of Chinese whispers?

Should the proponents of supply-chain repatriation and of renewed emphasis on import substitution retain the upper hand in the highest counsels of government, then analysts may want to reflect on the fragility of extant cross-border supply chains in sensitive sectors. They may also want to reflect on how such fragility came to pass despite the presence of global trade rules, an international organization to oversee them, and hundreds of regional trade agreements. Moreover, one might want to reflect on which international business models can thrive in the face of such fragility and intensifying geopolitical rivalry.

## Notes

1. Statements to this effect can be found in section 3.1 of this paper.
2. For an informative account of how historical analysis can contribute to the analysis of international business, see Buckley (2016).
3. In places, the author quotes at length from the statements of policymakers and from official reports. This was done because somehow the force of the arguments made is lost in the anodyne and restrained paraphrasing that is standard practice in academic writing.
4. In the interest of transparency, the author created the Global Trade Alert initiative.
5. Details of the goods collected and the list of trade policy interventions tracked by this tripartite initiative can be found in a methodology document obtained at https://www.globaltradealert.org/reports/54.
6. Note some policy interventions affect both food and medical goods sectors.
7. The ITC’s findings can be accessed here: https://www.macmap.org/covid19.
8. The other two organizations were the WTO and the World Customs Organization. Once the former geared up their monitoring, the latter ceased its useful work.
9. The United States also temporarily denied exporters of medical equipment access to trade finance from the U.S. Export-Import Bank, see https://www.globaltradealert.org/intervention/79199. Such a move discourages exports even if it does reduce the degree of subsidy-distorted competition in overseas markets.
10. Since Q1 2020 Chinese exports of PPE have rebounded.
11. See http://www.scio.gov.cn/zfbps/32832/Document/1681809/1681809.htm.
12. Comment made during a White House press conference on April 3, 2020. For video clip, see https://twitter.com/bennyjohnson/status/1245845521903882241.
13. Quoted in a Reuters article dated March 31, 2020 available at https://www.reuters.com/article/us-health-coronavirus-trade-ustr/coronavirus-shows-us-too-dependent-on-cheap-medical-imports-ustr-says-idUSKBN21I042.
14. Available at https://www.warren.senate.gov/imo/media/doc/2019.12.05%20Letter%20to%20DoD%20re%20pharmaceutical%20product%20supply%20chain.pdf.
15. Available at https://www.whitehouse.gov/wp-content/uploads/2017/12/NSS-Final-12-18-2017-0905.pdf.
16. Quoted in a news article in the South China Morning Post dated August 12, 2020 available at https://www.scmp.com/week-asia/opinion/article/3096911/coronavirus-has-complicated-china-japan-relations-how-will.
17. See https://ec.europa.eu/commission/sites/beta-political/files/communication-eu-china-a-strategic-outlook.pdf.
18. See https://www.bmwi.de/Redaktion/DE/Downloads/F/franco-german-manifesto-for-a-european-industrial-policy.pdf%3F__blob%3DpublicationFile%26v%3D2
19. Comments made at a press conference on April 2, 2020, available at https://www.gouvernement.fr/sites/default/files/locale/piece-jointe/2020/04/2108_-_bruno_le_maires_speech_-_international_press_conference_-_english_version.pdf.
20. As quoted in a Financial Times news article dated July 29, 2020, available at https://www.ft.com/content/80a4836b-ca25-48e0-996d-458186e968dc.
21. As quoted in a Reuters news article dated March 13, 2020 available at https://www.reuters.com/article/us-health-coronavirus-germany-pharmaceut/germany-would-like-to-localize-supply-chains-nationalization-possible-minister-says-idUSKBN2101BH.
22. For more details, see https://www.globaltradealert.org/intervention/79328.
23. See https://www.fema.gov/news-release/20200726/applying-defense-production-act.
24. See https://www.dfc.gov/media/press-releases/dfc-sign-letter-interest-investment-kodaks-expansion-pharmaceuticals.
25. See https://www.defense.gov/Explore/News/Article/Article/2319332/acquisition-enterprise-capabilities-to-continue-post-pandemic/
26. See the following “investments” by Canada’s Strategic Innovation Fund: https://www.canada.ca/en/innovation-science-economic-development/news/2020/08/government-of-canada-announces-major-steps-in-treating-and-preventing-covid-19-through-vaccines-and-therapies.html, and https://www.canada.ca/en/innovation-science-economic-development/news/2020/05/minister-bains-announces-investment-in-antibody-discovery-technology-to-help-treat-covid-19.html. Although framed in terms of supporting companies working on medical research, the official announcements also refer to investments in manufacturing capacity.
27. For more details, see https://www.globaltradealert.org/intervention/79270.
28. For more details, see https://www.globaltradealert.org/intervention/78924, https://www.globaltradealert.org/intervention/79006, https://www.globaltradealert.org/intervention/78923, and https://www.globaltradealert.org/intervention/79005.
29. For more details, see https://www.globaltradealert.org/intervention/79764 and https://www.globaltradealert.org/intervention/79762.
30. For more details, see https://www.globaltradealert.org/intervention/79598.
31. Consistent news reports indicate that in May 2020 the Korean government set aside 1.2 trillion Won (approximately $980 million) to develop that nation’s medical equipment sector; see https://en.yna.co.kr/view/AEN20200513001100320.
32. For more details, see https://www.globaltradealert.org/intervention/79860.
33. See https://www.chinadaily.com.cn/a/202007/27/WS5f1e0c65a31083481725c184.html.
34. Blanchette and Polk (2020) provide an alternative interpretation. Namely, that the dual circulation initiative amounts to a hedging strategy for Chinese policymakers.
35. For more information, see https://www.globaltradealert.org/state-act/44964/china-top-level-government-policy-released-by-state-council-to-encourage-exporters-to-sell-domestically.
36. For more information, see https://www.globaltradealert.org/state-act/44965/china-state-council-releases-top-level-policy-detailing-measures-to-safeguard-and-encourage-inbound-foreign-investment.
37. The notion of direct import dependence developed here can be distinguished from the vulnerability of a nation to a shock in a trading partner. The transmission mechanism of any such shock need not only affect bilateral trade flows.
38. This list of products and their respective product codes are available from the author upon request.
39. In this regard, it is noteworthy that calculations by U.S. Federal Reserve Bank of St. Louis using 2018 data on 18 categories of medical products revealed that the total foreign share of U.S. domestic absorption (a measure of consumption) was under 0.3. China’s share was less than 0.09 (Leibovici, Santacreu, & Peake, 2020). These estimates are not strictly comparable to those presented in the main text of this sub-section, as the St. Louis study used six-digit disaggregated import data.
40. Available at https://www.fda.gov/news-events/congressional-testimony/safeguarding-pharmaceutical-supply-chains-global-economy-10302019.
41. The statement is available here https://www.fda.gov/news-events/press-announcements/coronavirus-covid-19-supply-chain-update.
42. Statement available at https://www.fda.gov/news-events/press-announcements/coronavirus-covid-19-update-fda-and-cdc-take-action-increase-access-respirators-including-n95s.
43. Statement available at https://www.fda.gov/news-events/press-announcements/coronavirus-covid-19-update-fda-takes-further-steps-help-mitigate-supply-interruptions-food-and.
44. If anything, there may be pressures on firms from shareholders to strip out such capacity.
